# 

**Published:** 1878-01

**Authors:** 


*CONTENTS.*

| | Page | | Page |
|---|---|---|---|
| Men Wanted . . . . . . . | 351 | Retiring from Business . . | 358 |
| A Glass of Brandy . . . | 352 | Death of an Emperor . . . | 362 |
| Human Health . . . . . . . | 353 | Graham Bread . . . . | 365 |
| Preventable Society . . . | 354 | Hydrophobia . . . . . . . | 369 |
| Simples . . . . . . . . . . | 354 | Potatoes as Food . . . . | 370 |
| Sound Sleep . . . . . | 355 | Restless Wanderers . . . | 372 |
| Threatening of Disease . . | 356 | Vermin Riddance . . . . | 373 |
| The Good Physician . . . | 356 | Intussusception . . . . . . | 373 |
| Newspapers and Periodicals | 357 | Healthy Recreations . . . | 374 |
| Too White Flour . . . . | 358 | Going South in Winter . . | 375 |

